# An application of stereo matching algorithm based on transfer learning on robots in multiple scenes

**DOI:** 10.1038/s41598-023-39964-z

**Published:** 2023-08-06

**Authors:** Yuanwei Bi, Chuanbiao Li, Xiangrong Tong, Guohui Wang, Haiwei Sun

**Affiliations:** School of Computer Control and Engineering, Yantai University, Yantai, 264005 China

**Keywords:** Engineering, Electrical and electronic engineering, Mechanical engineering

## Abstract

Robot vision technology based on binocular vision holds tremendous potential for development in various fields, including 3D scene reconstruction, target detection, and autonomous driving. However, current binocular vision methods used in robotics engineering have limitations such as high costs, complex algorithms, and low reliability of the generated disparity map in different scenes. To overcome these challenges, a cross-domain stereo matching algorithm for binocular vision based on transfer learning was proposed in this paper, named Cross-Domain Adaptation and Transfer Learning Network (Ct-Net), which has shown valuable results in multiple robot scenes. First, this paper introduces a General Feature Extractor to extract rich general feature information for domain adaptive stereo matching tasks. Then, a feature adapter is used to adapt the general features to the stereo matching network. Furthermore, a Domain Adaptive Cost Optimization Module is designed to optimize the matching cost. A disparity score prediction module was also embedded to adaptively adjust the search range of disparity and optimize the cost distribution. The overall framework was trained using a phased strategy, and ablation experiments were conducted to verify the effectiveness of the training strategy. Compared with the prototype PSMNet, on KITTI 2015 benchmark, the 3*PE*-*fg* of Ct-Net in all regions and non-occluded regions decreased by 19.3 and 21.1% respectively, meanwhile, on the Middlebury dataset, the proposed algorithm improves the sample error rate at least 28.4%, which is the Staircase sample. The quantitative and qualitative results obtained from Middlebury, Apollo, and other datasets demonstrate that Ct-Net significantly improves the cross-domain performance of stereo matching. Stereo matching experiments in real-world scenes have shown that it can effectively address visual tasks in multiple scenes.

## Introduction

In recent years, robots have become essential assistants in various fields, including 3D scene reconstruction, target detection, autonomous driving, among others. The pervasive application of robotics technology across various industries has contributed to its integral role in modern life. Computer vision, a technology that emulates the human visual system and converts collected image information into target disparity information, plays a crucial role in assisting robots in accomplishing their tasks. Currently, a majority of robots rely on costly laser radar equipment to obtain high-precision disparity information. However, the principle of binocular vision, which closely replicates humans’ way of observing objects, is widely utilized in numerous visual tasks. The binocular stereo matching algorithm, a fundamental component of the binocular vision theory, directly impacts the accuracy of a robot’s target detection. By employing binocular vision theory, the robot can convert two-dimensional information into three-dimensional information of the target scene, thereby obtaining precise target scene information.

Stereo matching algorithms are crucial for understanding 3D scenes and reconstruction, and have been widely used in various fields, including robot navigation^1^, autonomous driving^2^, virtual reality^3^, and many others. These algorithms aim to calculate disparities, which represent the horizontal displacement of corresponding pixels in two rectified stereo pairs. Traditional methods often rely on prior knowledge of the image to construct a stereo matching function that enables the generation of a dense disparity map^4^.

Currently, convolutional neural networks (CNNs) are widely used in various vision tasks due to their powerful feature representation capabilities, including object detection^5^, image classification^6^, and more. In recent years, supervised stereo matching algorithms based on CNN have significantly improved the performance of stereo matching and have become the current mainstream research direction. The primary steps of the supervised stereo matching algorithm based on CNN include feature extraction, cost construction, and cost optimization.

However, the existing CNN-based stereo matching algorithms are primarily designed for fixed-structure models on specific datasets, while the issue of domain adaptive stereo matching has received limited attention from researchers. Moreover, previous studies have typically focused on obtaining network parameters through extensive training with large batches, disregarding the exploration of alternative training strategies. Kendall et al. were the first to proposed to obtain features through the ResNet^7^ structure and obtain disparity maps in an end-to-end manner. The domain adaptation module designed in DANet^8^ helps to reduce the domain shift. To enhance stereo matching performance, SegStereo^9^ incorporates a separately trainable semantic branch that provides disparity edge information for stereo matching. The optimization branch in this method employs a two-stage training process to eliminate redundant information and amplify matching-related information in concatenated volumes^10^. Nlca-net^11^ provides a bootstrap branch for optimizing disparity results. A semantic segmentation branch is proposed in work^12^ to incorporate additional semantic information into stereo matching tasks. PGNet^13^ proposed a panoptic parsing guided deep network to solve the stereo matching task. A cascading fusion cost volume is proposed to optimize the cost distribution^14^. Rao et al.^15^ enhanced the stereo matching performance of an existing model by implementing a new training strategy during retraining. Sang et al.^16^ proposed a spatial pyramid pooling attention module to address ill-posed areas and enhance the details of disparity maps through multi-scale context information capture. The above methods enhance stereo matching performance by optimizing the model’s structure and training strategy.

We present a novel stereo matching network that utilizes transfer learning and a customized training strategy to optimize the model. Firstly, we select a prototype network to provide improved parameter initialization for the stereo matching task. Next, to address the issue of inadequate feature learning, we employ a pre-trained model on large-scale datasets to extract general features. These features are then filtered to construct cost volumes that capture the similarity between stereo pairs. Furthermore, we train a feature adapter to enhance the screening capability of features for stereo matching, thereby minimizing the interference from non-stereo matching learning parameters. In contrast to existing algorithms that rely on single-scale features for cost construction, our approach incorporates a domain adaptive cost optimization module that replaces the original module in the prototype. Additionally, to further refine the cost volumes, we adjust the disparity range. Finally, we obtain the final disparity map through a regression method. In summary, there are three contributions in our paper:

- A domain adaptive stereo matching model for robots is proposed, which optimizes the stereo matching performance by grafting general features. Experiments conducted on multiple datasets and real-world scenes demonstrate that the model exhibits remarkable effectiveness across different domains.
- To capture general feature information, a grafted feature extractor is introduced and adapted to the network using a feature adapter. Additionally, an adaptive cost optimization module is introduced, and a disparity score prediction module is designed to adaptively adjust the disparity search range to optimize the cost distribution.
- A training strategy is proposed to train the prototype, feature adapter and domain adaptive cost optimization module, which provide better phased parameter initialization and update network parameters stage by stage, in addition, the training strategy of stereo matching is studied in this paper.

The paper is organized as follows. Section ”Related Works” presents the relevant background of stereo matching and introduces related work on traditional and deep-learning based algorithms for stereo matching. The implementation details of the proposed model (Ct-Net) are presented in Sect. ”Proposed Method”. Section ”Experimental Results and Discussions” provides details on the datasets used, experimental results, and discussions. Finally, the paper concludes with a summary and conclusion in Sect. ”Conclusion”.

## Related works

To date, robots have been widely applied in various fields and played an undeniable role. Shankar et al.^17^ proposed a passive stereo depth system consisting of CNN and a sensor that is designed to ensure the robot’s workspace. The proposed method was tested on multiple scenes and demonstrated effective application for home robots. Yang et al.^18^ proposed a probabilistic framework for robotic bin scene reconstruction systems that utilize active stereo camera data. Lajoie et al.^19^ presented the Swarm-SLAM system for collaborative simultaneous localization and mapping, which can be effectively applied to swarm robotics. Yang et al.^20^ proposed a CNN-based binocular vision self-inpainting network for real-time stereo image inpainting of autonomous robots, achieving state-of-the-art performance on image inpainting. Shim et al.^21^ proposed an inspection robot and management system that utilizes stereo vision to inspect damage on concrete surfaces. Obasekore et al.^22^ developed a recognition algorithm that utilizes a CNN-based binocular vision system in their agricultural robot to detect early-developmental pest stages in agriculture. Similarly, Xiang et al.^23^ proposed a field-based robot that utilizes binocular vision and CNN to detect and characterize the leaf angle of maize plants.

Stereo matching is a technique that allows for the recovery of depth information from stereo images. By simulating the principle of visual perception of human eyes, only two digital cameras placed on the same horizontal line are required. The main process of stereo matching includes image preprocessing, matching cost construction, cost aggregation, and disparity acquisition. Disparity, which refers to the horizontal displacement of spatial points in stereo pairs, is obtained through stereo matching. The goal of the stereo matching task is to accurately obtain a disparity map from a pair of corrected binocular images.

Traditional stereo matching algorithms include local algorithms, global algorithms, and semi-global algorithms. Local algorithms, such as correlation-based methods, have efficient implementations that make them suitable for real-time systems^24^. Compared to local algorithms, global stereo matching algorithms can calculate more accurate disparity by constructing a global energy function and minimizing the global cost^25,26^. However, the time required for global algorithms is relatively high. Furthermore, there is a semi-global stereo matching algorithm^27^ that calculates mutual information to measure the similarity of two images. It then uses dynamic programming to find the optimal matching path and minimize the global energy.

With the development of deep learning networks, stereo matching algorithms based on convolutional neural networks have emerged. Zbontar and LeCun^28^ were the first to introduce CNNs to calculating the matching cost and measuring the similarity between image patches. Luo et al.^29^ introduced a product layer in the Siamese network and proposed a multi-label classification network that calculates the local matching cost to enable multi-scale classification over disparities. Displets^30^ introduced image classification techniques to accurately determine object disparity. GC-Net^31^ introduced a 4D cost volume and used 3D CNNs to capture geometric and contextual information. PSMNet^32^ proposed the spatial pyramid pooling module to obtain the multi-scale features of images and introduced the hourglass structure for cost aggregation. GWC-Net^33^ proposed a group correlation strategy to construct a better matching cost volume by considering the correlation between different channel features. This approach enabled network to obtain a more accurate disparity map. A semantic segmentation branch was proposed in the SegStereo^9^ to incorporate additional semantic information to stereo matching tasks. PG-Net^13^ proposed a panoptic parsing guided deep network to solve the stereo matching tasks. PDSNet^34^ introduces a bottleneck matching module that enhances the ability to utilize global feature information. In addition, NLCA-Net-v2^15^ improved the stereo matching performance of the existing model by retraining with a new training strategy.

In recent years, research on domain adaptive models has become a hot topic. The network proposed in^35^ used different branches and cross-stage contextual information to exploit features at various resolutions, and proposed a branch cross-stage encoding module to regularize the cost volume. EdgeStereo^36^ explored the relationship between stereo and edge information in a unified learning model. HITNet^37^ introduced a fast multi-resolution initialization step and used a differentiable 2D geometric propagation and deformation mechanism to infer the disparity hypothesis. With the success of the Attention mechanism^38^ and Transformer^39^, some new methods such as CREStereo^40^ revisited stereo matching from new perspectives. Williem et al.^41^presented a deep self-guided cost aggregation method used to obtain an accurate disparity map from stereo images. Cheng et al.^42^ proposed convolutional spatial propagation networks (CSPN) based on spatial propagation networks (SPN), and extends CSPN to 3D for domain adaptive stereo matching tasks. Cheng et al.^43^ incorporated geometric knowledge into the neural architecture search framework and proposed LEAStereo. It is the first end-to-end hierarchical NAS framework for deep stereo matching. A cost network based on cascade and fusion is proposed to improve the robustness of stereo matching^14^. DSMNet^44^ is designed with two novel trainable neural network layers that generalize well across domains without fine-tuning or domain adaptation.

In this work, we designed our network structure based on PSMNet^32^ and ResNet^7^. In addition, we used transfer learning techniques to introduce general domain features into stereo matching tasks. In order to obtain a better matching cost, we designed a domain adaptive cost optimization module that can adaptively adjust the disparity search range. Meanwhile, a training strategy was used to integrate the above modules into our stereo matching framework. More details are described below.

## Proposed method

**Figure 1:**
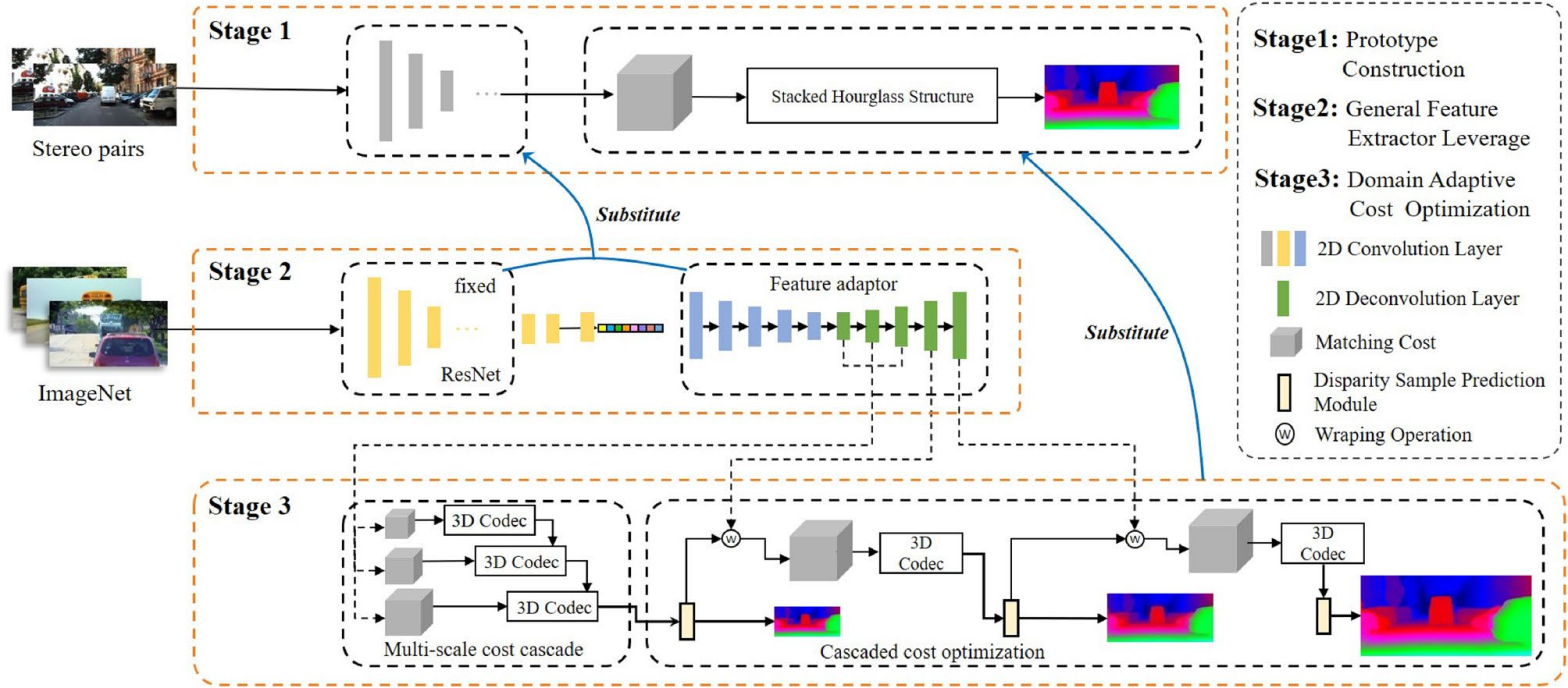
Overview of the network architecture and training strategy. The network consists of three training stages. In the stage 1, we use PSMNet as the prototype, and the trained prototype as the initialized parameters in the next stage, and then the model parameters are updated step by step. In the stage 2, we introduce a General Feature Extractor (GFE) consists of part of the ResNet model pretrained on ImageNet and a Feature adapter to substitute the feature extractor in the prototype, and then train the improved network. In the stage 3, based on the trained model in the stage 2, we substitute the Stacked Hourglass Structure in the prototype with the Domain Adaptive Cost Optimization Module (DACOM), and use the optimal model in the stage 2 as the initialization parameter, then train the final improved model and get accurate disparity map.

A deep-learning network trained in stages is proposed for the stereo matching task in this paper, and the overall structure is shown in Fig. 1. In the first training stage, a prototype is constructed as the initial structure of the network to provide parameter initialization for the subsequent stage. In the second training stage, the original feature extraction structure is replaced with the general feature extractor (GFE), which is pretrained on ImageNet^45^, and a U-shaped feature adapter is trained to adapt the pre-trained features to the cost optimization module of the network. In the third stage, the cost optimization in the prototype is replaced with a domain adaptive cost optimization module (DACOM) to get a better disparity map. Model training is co-supervised using the Smooth l1 loss along with the Mean Absolute Error (MAE) loss through multiple stages.

### Prototype construction

As the first stage of the model training process, the prototype can provide better parameter initialization for the next training stage. Therefore, selection of the prototype is critical. The PSMNet exhibits an excellent stereo matching effect while maintaining a relatively straightforward structure^32^, so we use the overall structure of PSMNet as the prototype.

After the first training stage is completed, the learned parameters of the cost optimization module in the prototype are fixed for training of the feature adapter in the second stage.

### General feature extractor leverage

The general feature extractor (GFE) is a key component of the network structure. Extracting general features is very important for the performance of the domain adaptive stereo matching network, and we need select a model pre-trained on a large-scale dataset to alleviate domain shift. The model can learn various styles of images on large-scale datasets such as the ImageNet dataset. Therefore, this work is beneficial for domain adaptive tasks. Our algorithm selects the powerful ResNet-18 model pre-trained on ImageNet as the backbone structure of the GFE and fixes its parameters in the public domain feature extractor, as shown in Fig. 1. The grafted ResNet^7^ model can further extract wide-domain feature information from the shallow features. Unlike the classification task, the model only uses the structure before the fourth down-sampling of the ResNet^7^ model to extract features at 1/2, 1/4, and 1/8 scales of the original image size, respectively. The basic structure includes 3$\times$3 Convolutional layers (Conv), Batch Normalization (BN) layers , ReLU activation functions, and down-sampling (Max-pooling) layers.

Similar to grafting trees, a suitable interface is necessary to ensure the provided features can be effectively used in the next stage. Therefore, we also incorporate a feature adaptor to further refine the features for optimal compatibility with the network. After the prototype training stage, the grafted model is also utilized in the second stage to extract features using GFE. Since the grafted model has been trained on ImageNet, it possesses generalization abilities that are beneficial for domain adaptive stereo matching, not being specifically trained on large-scale stereo datasets. Furthermore, the feature adapter can effectively eliminate redundant information and enhance stereo-task information for matching cost construction in the subsequent training stage.

### Domain adaptive cost optimization

The features obtained from GFE contain rich semantic information, requiring further processing with a deeper network structure. Moreovers, the regression or classification by constructing the single scale cost may lead to redundant or insufficient feature information, the model may be overfitting on a certain domain, and the robustness of the algorithm may be affected. As explained in related works^46–48^, multi-scale feature information can be utilized to obtain multiple receptive fields. Jeon et al.^46^ proposed an efficient multi-scale sequential feature fusion network to fully regularize the cost volume. MSCVNet^47^ first generates multiple 3D cost volumes with different resolutions for cost aggregation. A multi-scale pyramid aggregation module is designed to effectively utilize the aggregation information of different scales^48^. Therefore, Ct-Net employs multi-scale features obtained from the corresponding up-sampling stage of the feature adaptor as input to construct multi-scale cost volume. Specifically, the multi-scale matching cost volumes are constructed by group correlation method^33^ proposed by Guo et al.The basic idea of the group correlation matching cost construction method is as follows: First, the features are grouped and the correlation mapping is calculated group by group. The feature channel is represented as ${N_{c}}$. All features are divided into ${N_{g}}$ groups along the channel dimension. The calculation formula of group correlation can be expressed as follows,

1 $$\begin{aligned} C_{g w c}(d, x, y, g)=\frac{1}{N_{c} / N_{g}}<f_{l}^{g}(x, y), f_{r}^{g}(x-d, y)> \end{aligned}$$

where $<,>$ represents the inner product operation, and the correlation of features is calculated for the feature group *g* and all disparity levels *d*.

**Figure 2:**
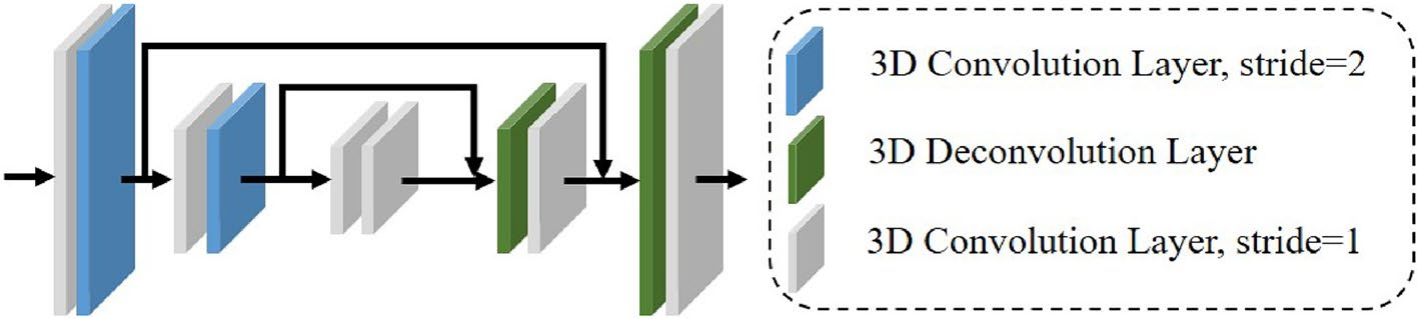
3D codec structure.

Due to the influence of the ill-posed regions, the initial cost contains massive noise information. The noise information of multi-scale costs is further filtered out by 3D codec. The 3D codec mainly includes 3D convolution layers and 3D deconvolution layers. Figure 2 shows the main structure of the 3D codec. Additionally, we cascade the filtered multi-scale costs to increase the interaction of multi-scale information. Specifically, the high-scale cost merged with the up-sampled low-scale cost using the addition operation, which increases semantic information acquisition and reduces the loss of detailed information.

The cost reflects the matching similarity between candidate pixels. However, the cost distribution of pixels is often multimodal, as shown in the low-scale cost of Fig. 2. This can result in a high disparity error. To alleviate the above problem, after the fusion of three matching costs from low to high, we adjust the next cost distribution by predicting the disparity samples. First, we predict the disparity score for each spatial point, which is then used as input for constructing the last two matching costs. The formula of the disparity score prediction is as follows:

2 $$\begin{aligned} \left\{ \begin{array}{l} {\hat{d}}=\sum _{\forall d} d \times \sigma \left( -c_{d}\right) \\ F_{\textrm{score}}=\sum _{\forall d}|d-\hat{d}| \times \sigma \left( -c_{d}\right) \end{array} \right. \end{aligned}$$

among them, $\hat{d}$ represents the predicted disparity, *d* represents the candidate disparity, $\sigma$ represents the softmax operation and $c_{d}$ represents the matching cost. The disparity search range of the next stage can be adjusted according to the disparity score. The disparity search range of each point (*i*, *j*) in the next stage can be expressed as:

3 $$\begin{aligned} \left\{ \begin{array}{l} d_{\min }(i, j)=\hat{d}(i, j)-\alpha F_{\text{ score } }(i, j) \\ d_{\max }(i, j)=\hat{d}(i, j)+\alpha F_{\text{ score } }(i, j) \end{array}\right. \end{aligned}$$

$\alpha$ is initialized to 1, which can be learned by the network.

Due to the different scales of the predicted disparity score map, the obtained disparity range maps are respectively up-sampled by bilinear interpolation. After that, we obtain disparity samples of each point as the input of the next step by uniform sampling between $d_{\min }$ and $d_{\max }$ , the disparity samples can be expressed as:

4 $$\begin{aligned} d_{\text{ sample } }=d_{\min }(i, j)+\frac{S}{S-1}\left( d_{\max }(i, j)-d_{\min }(i, j)\right) \end{aligned}$$

among them, *S* represents the disparity samples size of point (*i*, *j*) and $s \in (0,1,2, \ldots , S-1)$ . We fuse the disparity samples with the right feature map using a wrapping operation^49^, and then construct the matching cost using the group correlation method. This cost is optimized using the 3D codec.

Finally, we use the last disparity sample prediction module to obtain the final disparity image.

### Evaluation metrics

In order to quantitatively evaluate the performance of our algorithm, we evaluate the proposed algorithm using *xPE*, where *xPE* represents the percentage of pixels for which the predicted disparity is off by more than *x* pixels, and *EPE* refers to the average difference between the predicted disparity and the ground truth.

The evaluation metrics can be expressed as follows:

5 $$\begin{aligned} x P E&=\frac{1}{N} \sum _{(i, j)}\left( \left| \hat{d}(i, j)-d^{*}(i, j)\right| _{1}>x\right) \times 100 \% \end{aligned}$$

6 $$\begin{aligned} E P E&=\frac{1}{N} \sum _{(x, y)}\left( \left| \hat{d}(i, j)-d^{*}(i, j)\right| _{1}\right) \end{aligned}$$

among them, *N* represents the total number of pixels, $\hat{d}$ and $d^{*}$ represents the predicted disparity and ground truth of pixel, respectively.

## Experimental results and discussions

In this study, the proposed algorithm is implemented using PyTorch framework, trained and tested on a single NVIDIA Tesla V100 GPU with batch size set to 2. The Adam optimizer was used, and the parameters were set to $\beta _1$ =0.9 and $\beta _2$ =0.999. Scene Flow^50^ is used as the pre-training dataset, and KITTI^51^, Middlebury^52^, and Apollo^53^ are used to verify the performance of the algorithm.

**Table 1:** Model ablation experiments with different training strategies.

Training strategy	Scene flow	
	3*PE*(%)	*EPE*(px)
Model of stage 1	2.87	1.09
Model of stage 2	2.80	0.97
Model of stage 2 (stage 1)	2.75	0.90
Model of stage 3	2.65	0.84
Model of stage 3 (stage 2)	2.53	0.71
Model of stage 3 (stage 1, stage 2)	**2.45**	**0.67**

Model of stage i, Model of stage j means that the j-stage model has been trained by the i-stage model, and the optimal model of the i stage is used as the initialization parameter.

Significant values are in [bold].

### Dataset description

In the experimental part, we use SceneFlow, KITTI, Middlebury and Apollo datasets to train and test the model.

Scene Flow^50^: It is a large synthetic dataset with an image size of 960$\times$540 px, including 35,454 training image pairs and 4370 test image pairs. It provides the ground truth of disparity and the maximum disparity is 192. network training takes about 50 hours for 10 epochs, and the learning rate is set to 0.001.

KITTI^51^: Including KITTI2012 and KITTI2015, is a challenging and diverse road scene dataset with a size of 1236$\times$376 px, and only a sparse disparity map is provided as the training standard. We fine-tuned the model on these two data sets. It takes about 48 hours to train the network for 300 epochs, and the learning rate is set to 0.001 for the first 200 epochs and 0.0001 for the last 100 epochs.

Middlebury^52^: A small indoor dataset used to verify the generalization ability of the model for real scenes. The image is divided into three scales: F, H and Q. The data of scale Q is used for verification, and the maximum disparity is 256.

Apollo^53^: The Apollo dataset consists of 5165 image pairs and corresponding disparity maps, of which 3324 image pairs are used for training, 832 image pairs are used for validation, and 1009 image pairs are used for testing. Ground truth has been obtained by accumulating 3D point clouds from lidar and separately acquiring a dataset of 3D car instances. This dataset contains different traffic situations with severe occlusion, which is challenging.

For each stage, the SceneFlow dataset is used as a pre-training dataset to train the model because it contains many images and scenes, while the Middlebury, KITTI, and Apollo datasets are relatively small and test the model’s performance after model fine-tuning.

### Analysis of experimental results

We conduct ablation studies on the training strategy and algorithm modules on the above five datasets.

**Figure 3:**
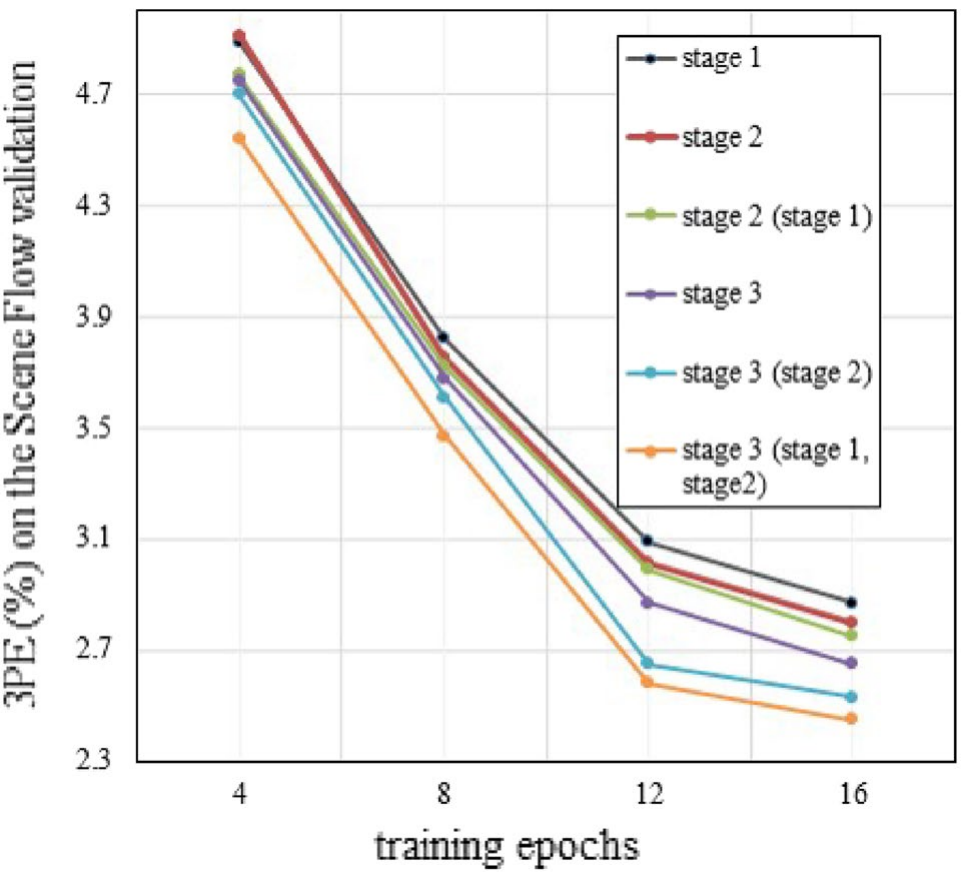
Convergence process of models with different training strategies. (stage x) means the pre-training model of stage x. It shows that the staged training strategy can decrease the matching error rate compared with end-to-end training strategy, and reasonable models can increase the upper limit of final’s results.

First, we use the Scene Flow dataset to verify the impact of the training strategy on the model. The results of the ablation experiments are shown in Table 1. Compared with the model trained directly in stage 2, the 3*PE* and *EPE* are decreased when the model is trained in the second stage and pre-trained in stage 1. At the same time, compared with the model trained directly in stage 3, the model trained in stage 3, which pretrain in stage 1 and stage 2, 3*PE* and *EPE* metrics decline by 0.20% and 0.17px, respectively. The above ablation experiments show that a staged training strategy is helpful in improving model performance. Figure 3 shows the convergence process of different training strategies. Compared with the end-to-end model that was trained solely in stage 3, the model trained using the strategy of stage 3 (stage 1, stage 2) was better in terms of accuracy at different epochs. Furthermore, compared with the prototype in stage 1, the model in stage 2 showed a decrease in 3*PE* and *EPE* metrics, thereby verifying that the general feature extractor can improve the performance of stereo matching. These experiments show that different training strategies affect the performance of the final model. The ablation studies were conducted on different modules, and the results of the experiments are as follows.

**Table 2:** Experimental results of different network settings on multiple datasets.

Experiment	Method	KITTI		Middlebury	
		3*PE*(%)	*EPE*(px)	3*PE*(%)	*EPE*(px)
Feature	Feature extractor of prototype	4.6	0.89	22.93	5.94
Extraction	General feature extractor	3.9	0.83	22.65	5.83
Cost	Stacked hourglass structure of prototype	5.3	0.94	22.63	5.85
Optimization	Domain adaptive cost optimization module	3.5	0.82	22.01	5.35
Disparity	Not fused disparity samples	4.7	0.88	23.96	5.96
Sample	Fused disparity samples	3.4	0.76	22.83	5.23
Loss	Smooth L1 loss	4.6	0.89	23.86	5.85
Function	Smooth L1 loss + MAELoss	4.3	0.88	22.83	5.33

The evaluation metrics are 3*PE* (%) and *EPE* (px).

**Figure 4:**
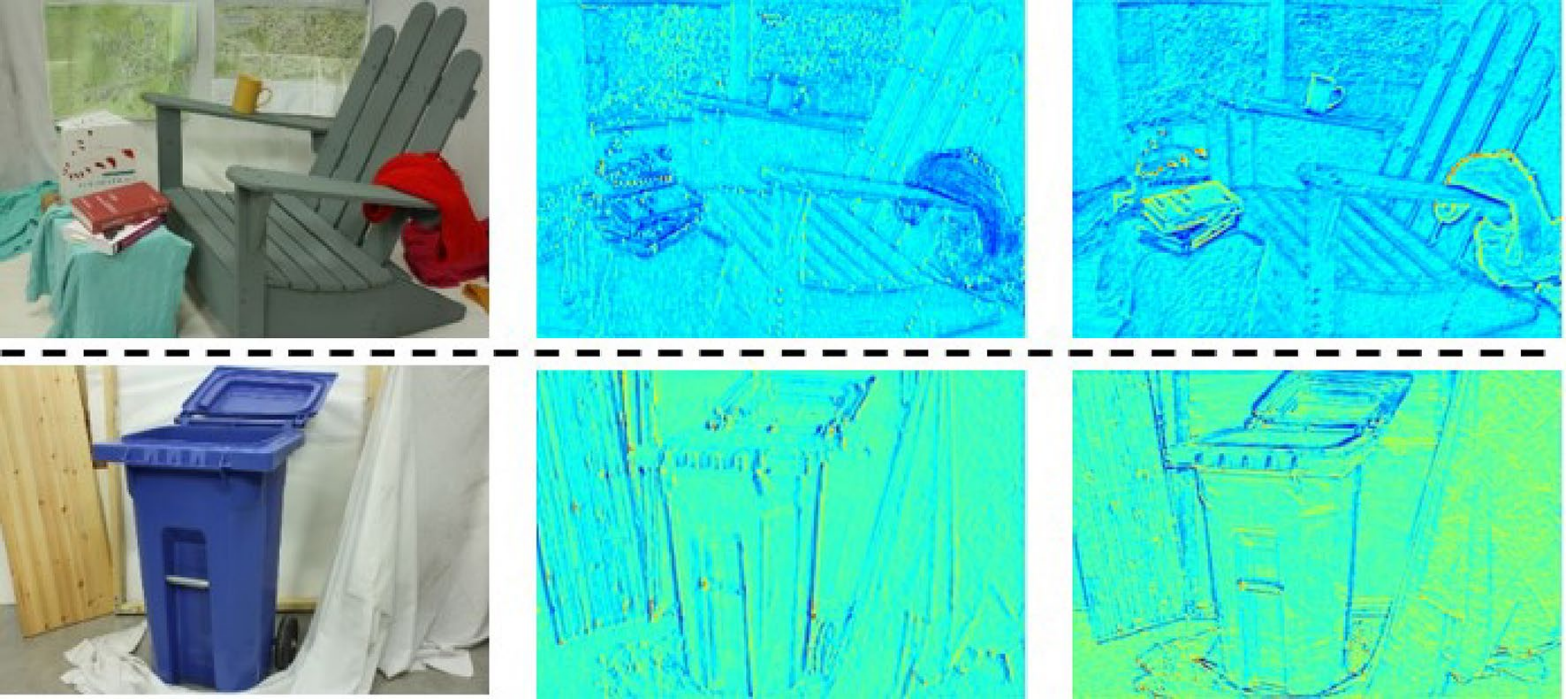
Comparison of feature visualization samples obtained by feature extractor of prototype and GFE. From left to right, left image, the feature acquired by feature extractor of prototype, and the features acquired by GFE.

We compare the prototype with the grafted ResNet model mentioned in this paper. It can be seen from Table 2 that for the KITTI dataset, the 3*PE* and *EPE* metrics of the model with General Feature Extractor (GFE) drops from 4.6% and 0.89px to 3.9% and 0.83px, respectively. And for Middlebury datasets, the algorithm accuracy of GFE is also slightly improved. At the same time, since the parameters of the ResNet module in GFE have been pre-trained on the ImageNet dataset, and the parameters are fixed, there is no need to update the parameters during the model training stage, which relatively improves the efficiency of the model.

The qualitative analysis of the features acquired by different feature extractors was conducted. The feature visualization samples are shown in Fig. 4. It can be found that there are obvious differences between the two features obtained by the feature extractor of prototype and GFE. The latter contains more semantic and texture information, which is seen as the key information to deal with the high matching error rate of ill-posed regions. Both quantitative and qualitative results show that the GFE is beneficial for stereo matching tasks.

The ablation experimental results of domain adaptive cost optimization module are shown in Table 2, which shows that the domain adaptive cost optimization module (DACOM) can achieve better performance than the stacked hourglass structure of prototype. Specifically, on the KITTI dataset, compared with the stacked hourglass structure of prototype, the 3*PE* and *EPE* of the model with DACOM decrease from 5.3% and 0.94 to 3.5% and 0.82. Meanwhile, for Middlebury dataset, the 3*PE* and *EPE* metrics of DACOM decrease from 22.63% and 5.85–22.01% and 5.35, respectively. The quantitative results show that the adaptive cost optimization strategy achieves better performance.

**Figure 5:**
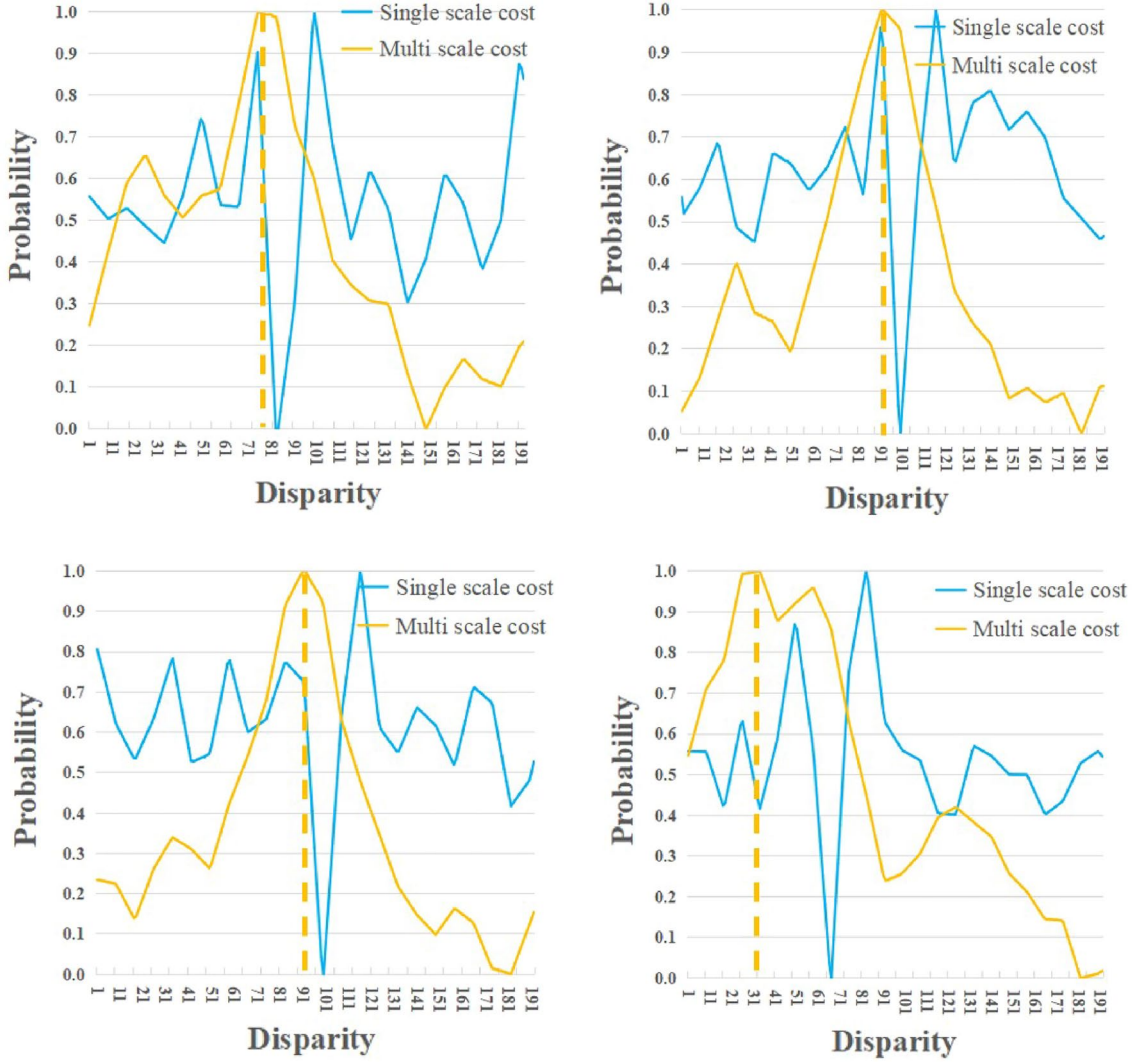
Cost distribution of multi-scale costs. As the cost scale increases, the cost distribution gradually tends to be unimodal distribution and the peak is near the ground truth.

Additionally, we conducted ablation experiments on the multi-scale cost cascade strategy, and the experimental results are shown in Table 3. From the results, it can see that as the multi-scale cost increases, 3*PE* and *EPE* metrics decrease simultaneously. Specifically, for Scene Flow, compared with only high scale cost used, 3*PE* and *EPE* metrics of (high, medium, and low costs) were decreased by 6.8% and 0.05px. To further explore the role of multi-scale cost in stereo matching tasks, the contrast experiment is set up and the results are shown in Fig. 5. From the results, it can see that the cost distribution tends to be multimodal at a single-scale cost (The solid blue line in Fig. 5), which is not beneficial to obtain optimal disparity results by matching costs. When we visualize the multi-scale cost, the cost distribution tends to be the unimodal distribution (The solid yellow line in Fig. 5), and the optimal cost value tends to the disparity ground truth (The disparity value corresponding to the yellow dotted line in Fig. 5). It can be deduced from the quantitative and qualitative results that multi-scale costs can reduce false matching due to distribution. We hypothesize that since the input image contains ill-posed regions, inaccurate initial low-scale matching cost often leads to matching errors and irreversible results, and the supplementary multi-scale information optimizes above phenomenon.

**Table 3:** Ablation experiments of the three multi-scale costs of the domain adaptive cost optimization module. We calculated 3*PE* and *EPE* on the Scene Flow and KITTI verification sets, respectively.

Experiment setting	Scene flow		KITTI	
	3*PE*(%)	*EPE*(px)	3*PE*(%)	*EPE*(px)
Only high scale cost	2.63	0.72	4.60	0.83
High and medium scale costs	2.56	0.70	4.37	0.78
High, medium and low scale costs	**2.45**	**0.67**	**4.25**	**0.77**

Significant values are in [bold].

**Figure 6:**
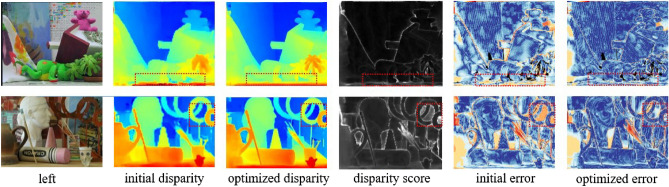
Comparison of initial disparity, initial error, disparity score, optimized disparity, and optimized error. The binocular image from https://vision.middlebury.edu/stereo/. The error map tends to be warmer color to indicate a higher error rate. False disparity is always present on the ground or at the edge of objects, and the corresponding disparity score is relatively high in these areas. After adjusting for the disparity score, warm colors are significantly reduced in error map and the disparity edges becomes smoother.

As discussed above, matching costs are closely related to the disparity results, so how to further optimize matching cost become the key step. We use the Disparity Sample Prediction module to adaptively adjust the candidate disparity range before creating the matching cost. The ablation experiment results are shown in Table 2. From the results, it is evident that fusing the disparity samples before cost construction leads to a decrease in both 3*PE* and *EPE* metrics for Scene Flow and KITTI datasets. This suggests that adding disparity samples can improve the performance of stereo matching. Furthermore, as the disparity search range of each spatial point requires prediction before generating the disparity sample, and the generation of the disparity search range is based on the predicted disparity score, we visualize both the disparity score map and error map. The visualization results are presented in Fig. 6. As can be observed from the figure, regions with high disparity scores always exhibit higher errors, suggesting a close relationship between the disparity score and the regions that require optimization. The disparity map and error map optimized using the disparity score are superior to the initial disparity map and error map, highlighting the disparity adjustment capability of the domain adaptive cost optimization module.

**Table 4:** Ablation experiments of the disparity samples size S and stereo matching performance.

Setting of S	Scene flow		KITTI	
	3*PE*(%)	*EPE*(px)	3*PE*(%)	*EPE*(px)
S=10	2.50	0.72	4.33	0.81
S=20	2.47	0.70	4.27	0.78
S=30	**2.46**	**0.68**	**4.25**	**0.77**

S” represents the size of the disparity sample generated by the disparity sample prediction module.

Significant values are in [bold].

Furthermore, we set up ablation experiments to verify the relationship between disparity samples size *S* and stereo matching performance. The results are shown in Table 4, when *S* gradually increases, the stereo matching performance will gradually increase. This is also in line with common sense that the more disparity samples, the higher of the disparity accuracy. Weighing the time consumed by the network and accuracy, we set *S* to 30 in this paper. In summary, the Domain Adaptive Cost Optimization Module can optimize the cost distribution and further optimize the performance of stereo matching.

Finally, we conducted an ablation experiment on the loss function, and the results are shown in Table 2. Mixing the MAE loss function has better results than using only the Smooth L1 loss function.

**Table 5:** Comparing experimental results of our method with other methods on the KITTI 2012 benchmark.

Method	$2PE-all$	$3PE-all$	$4PE-all$	$5PE-all$	*EPE*
SGM	10.16	7.00	5.41	4.41	1.3
PVStereo	5.25	2.47	1.70	1.33	0.8
PSMNet	3.01	1.89	1.42	1.15	0.6
PDSNet	4.65	2.53	1.85	1.51	1.0
SegStereo	3.19	2.03	1.52	1.21	0.6
HSM	3.32	1.99	1.46	1.16	0.6
AANet+	2.96	2.04	1.58	1.30	0.5
CFNet	*2.43*	*1.58*	*1.18*	*0.94*	0.5
LEAStereo	**2.39**	**1.45**	**1.08**	**0.88**	**0.5**
Ours(Ct-Net)	2.91	1.97	1.54	1.29	0.6

Significant values are in [bold and italic].

Based on the above discussions, we can conclude that the proposed modules and training strategy are effective in improving the performance of stereo matching.

### Cross-domain generalization performance

One of the primary challenges in cross-domain stereo matching is the domain shift problem. This issue arises when a model trained on one domain (or dataset) performs poorly when applied to a different domain due to variations in image characteristics, such as lighting conditions, camera parameters, and scene compositions.

In this section, to verify the cross-domain generalization performance of the algorithm, we selected the Middlebury, KITTI, and Apollo datasets as the test set and the Scene Flow dataset as the training set.

#### Experiments on the KITTI 2012 and KITTI 2015 dataset

The comparison results are presented in Tables 5, 6, 7, and 8. The final submission results on the KITTI benchmark are shown in Tables 5 and 6, and the evaluation metrics are *xPE* percentage for all regions and non-occluded (Noc) regions. In the KITTI 2012 benchmark, the proposed algorithm showed a significant improvement in *xPE* percentage compared to the traditional algorithm SGM^27^. Additionally, when compared to the high-precision deep learning algorithm AANet+^54^, which efficiently performs cost aggregation using sparse point-based feature representation, the proposed algorithm demonstrated lower *xPE* in all regions. Compared to other deep learning-based stereo matching algorithms, such as PVStereo^55^, PDSNet^34^, SegStereo^9^, and HSM^56^, the proposed algorithm achieved the lowest *xPE* percentage. However, when compared to the state-of-the-art methods CFNet and LEAStereo^43^, the proposed algorithm still performed relatively poorly.

**Table 6:** Comparing experimental results of our method with other methods on the KITTI 2015 benchmark.

Method	All regions(%)			Nocregions (%)		
	$3PE-bg$	$3PE- fg$	$3PE-all$	$3PE-bg$	$3PE-fg$	$3PE-all$
SGM	5.06	13.00	6.38	4.43	11.68	5.62
PVStereo	2.29	6.50	2.99	2.09	5.73	2.69
PSMNet	1.86	4.62	2.32	1.71	4.31	2.14
PDSNet	2.29	4.05	2.58	2.09	3.68	2.36
SegStereo	1.88	4.07	2.25	1.76	3.70	2.08
HSM	1.95	3.93	2.28	1.76	3.55	2.06
AANet+	1.65	3.96	2.03	1.49	3.66	1.85
CFNet	1.54	3.56	1.88	1.43	3.25	1.73
LEAStereo	**1.40**	**2.91**	**1.65**	**1.29**	**2.65**	**1.51**
Ours (Ct-Net)	1.85	3.73	2.16	1.69	3.40	1.97

“Noc” means non-occluded area; “fg” represents the foreground area; “bg” represents the background area; “all” represents the all area.

Significant values are in [bold].

**Table 7:** Comparing experimental results with other methods on the Middlebury dataset.

Method	AustraliaP	Bicycle2	Crusade	CrusadeP	DjembeL	Livingroom	Staircase
FADNet	9.48	13.9	43.4	45.0	57.8	24.8	68.1
PSMNet	62.3	53.4	60.4	54.1	52.6	54.5	34.1
AANet	11.3	12.9	33.4	30.9	28.8	25.3	69.8
iResNet	9.19	15.8	27.7	16.8	54.7	19.5	51.6
CFNet	7.81	7.12	12.3	11.5	3.02	10.7	9.01
LEAStereo	**4.52**	**4.62**	**5.86**	**6.03**	**3.30**	**11.3**	**9.90**
Ours (Ct-Net)	11.3	11.7	27.1	20.4	27.9	16.5	24.4

The evaluation metrics is 2*PE* (%).

Significant values are in [bold].

**Table 8:** Experimental results of our method compared with the prototype on the Apollo dataset.

Method	$3PE-bg$	$3PE-fg$	$3PE-all$	*EPE*
PSMNet	5.25	5.98	3.31	0.89
Ours (Ct-Net)	**3.31**	**3.81**	**2.89**	**0.74**

Significant values are in [bold].

**Figure 7:**
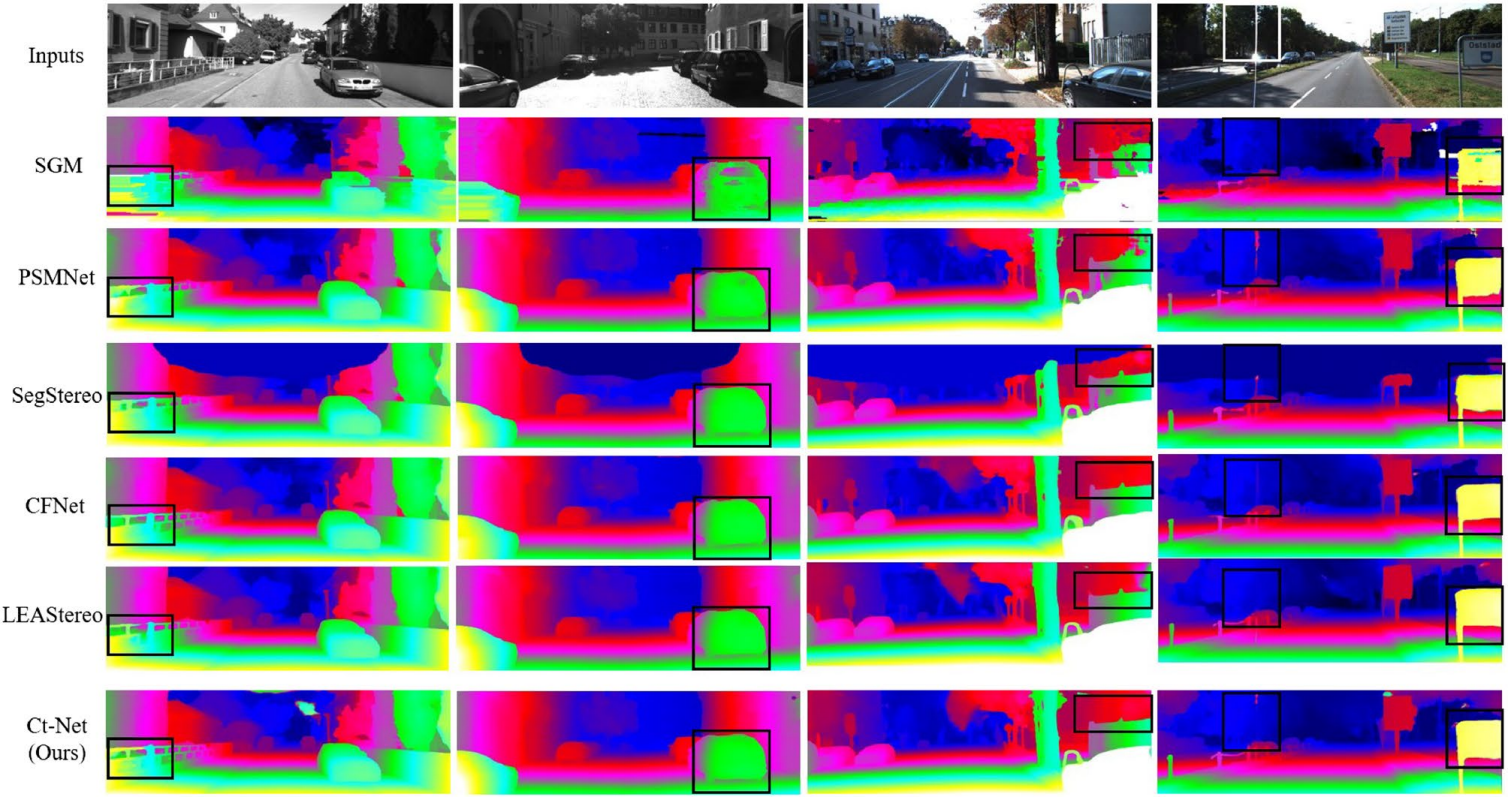
Qualitative results of KITTI benchmark. In this article, we compared our method with disparity maps of other algorithms. The left two columns are KITTI2012 samples, and the right two columns are KITTI2015 samples. The black box in the image is the area with obvious difference.

In addition, as shown in the black box in Fig. 7, we can achieve better disparity prediction on image detail and overall target structure, and produce a smoother disparity image compared to SGM^27^. Compared with PSMNet^32^, although the disparity effect generated by SGM is better than that of the traditional algorithm, the algorithm can not produce correct disparity results in areas such as car Windows, and the algorithm proposed in this paper achieves better results on car Windows. SegStereo^9^ introduces image edge information to improve the disparity edge effect. Compared with SegStereo, the proposed algorithm achieves better results in fence railings and vehicle chassis. CFNet^14^ uses multi-scale cost optimization to obtain better disparity results. Compared with CFNet, the proposed algorithm achieves a comparable effect in disparity detail region. In addition, compared with LEAStereo^43^, which performs well in recent years, the disparity results produced by this algorithm also perform well on the road. Benchmark test results by KITTI 2012 show that the performance of this algorithm is comparable to that of existing advanced algorithms.

**Figure 8:**
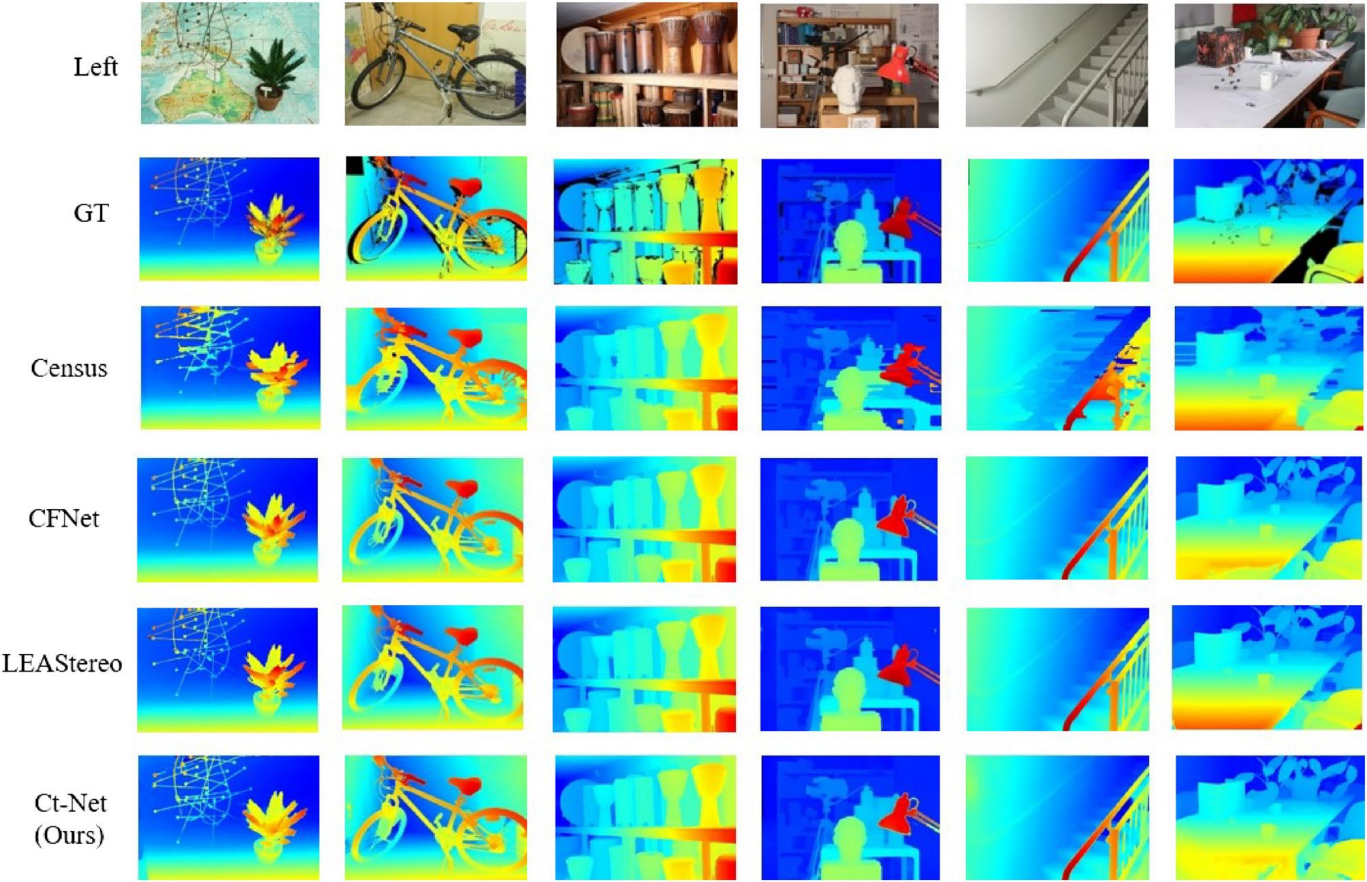
Qualitative results of the Middlebury dataset. The binocular image from https://vision.middlebury.edu/stereo/. From top to bottom, the left image, the ground truth GT, the disparity maps of Census, the disparity maps of FADNet, the disparity maps of iResNet, the disparity maps of Ct-Net (Ours).

**Figure 9:**
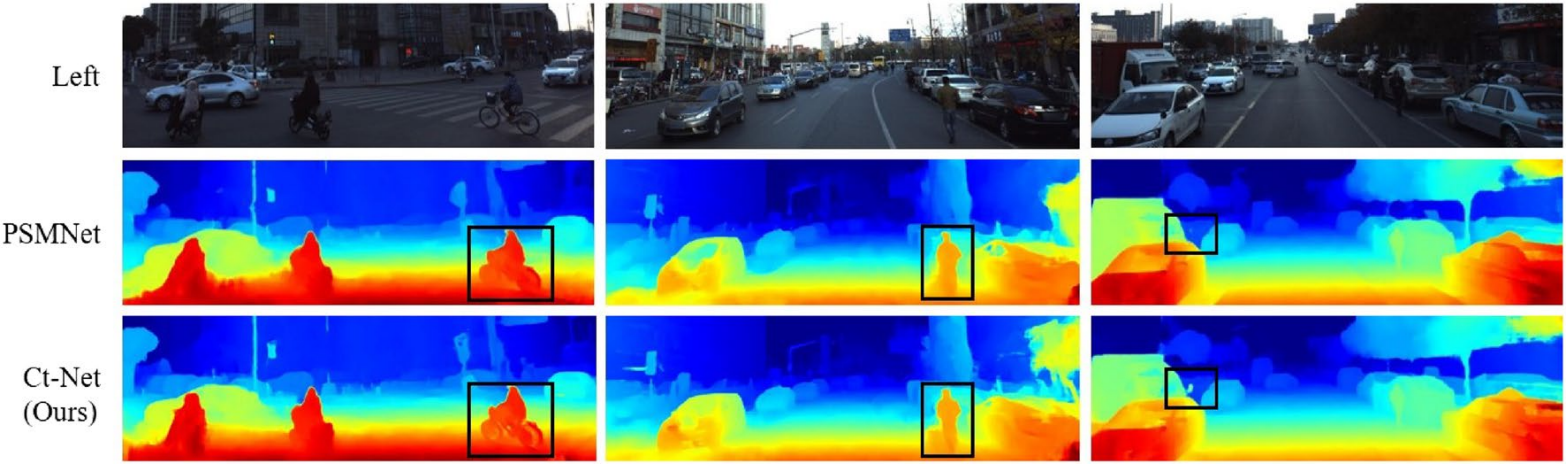
Qualitative results of Apollo test dataset. The binocular image from https://apolloscape.auto/stereo.html. The first row is the left images, the second row is the PSMNet disparity maps, and the third row is the predicted disparity maps by our network. The black box in the image is the area with obvious difference.

In the KITTI 2015 benchmark, when compared to the prototype PSMNet^32^, CtNet showed a significant improvement with a decrease of 19.3 and 21.1 % in $3PE-fg$ for all regions and non-occluded regions, respectively. Furthermore, the proposed algorithm achieved a lower *xPE* percentage compared to others. Compared with the high-precision deep learning algorithm AANet+^54^, the proposed algorithm is improved in $3PE-fg$ metric on all regions and non-occlusion regions. In addition, compared with other deep learning based stereo matching algorithms, such as PVStereo^55^, PDSNet^34^, SegStereo^9^ and HSM^56^, the proposed algorithm obtains the lowest *xPE* percentage. The qualitative results of the KITTI 2015 benchmark are shown in Figure 7. Our algorithm achieves more detailed and accurate predictions compared to SGM. Compared with the PDSNet^34^, SegStereo^9^, HSM^56^, the proposed algorithm achieves better results on fence railings and highway street signs. Similarly, compared to state-of-the-art algorithms CFNet and LEAStereo, the proposed algorithm still has room for improvement. However, qualitative and quantitative results on the KITTI 2015 benchmark demonstrate that our algorithm is well-suited for stereo matching tasks in road scenes.

#### Experiments on the middlebury dataset

The test results on Middlebury benchmark are shown in Table 7. Compared with the method based on deep learning, such as FADNet^57^, PSMNet^32^, and AANet^54^, the proposed method has a lower error rate on all samples. Compared with the high-precision deep learning algorithm iResNet^58^, the proposed algorithm performs better on the samples Bicycle2, Crusade, DjembeL, Livingroom, and Staircase. Compared with other samples, the difference in error rate is slight. Furthermore, the qualitative results are shown in Fig. 8, where we compare different methods on six samples from Middlebury. Compared with the traditional method Census^24^, the proposed algorithm attains better disparity edge stereo matching performance and improves the detection performance of ill-posed regions such as thin structures and texture-less regions. Compared with the method PSMNet^32^ based on deep learning, the proposed algorithm has a better stereo matching performance on details.

#### Experiments on the apollo dataset

Finally, we compared the proposed algorithm with PSMNet^32^ on the Apollo dataset. As shown in Table 8, our algorithm outperforms PSMNet^32^ in all metrics. The qualitative results are shown in Fig. 9. Compared with PSMNet^32^, the algorithm in this paper has better stereo matching performance in detail areas such as bicycles and pedestrians.

The qualitative and quantitative analysis results show that the proposed algorithm achieves promising results on multiple datasets.

### Experiments for real-world scenes

**Figure 10:**
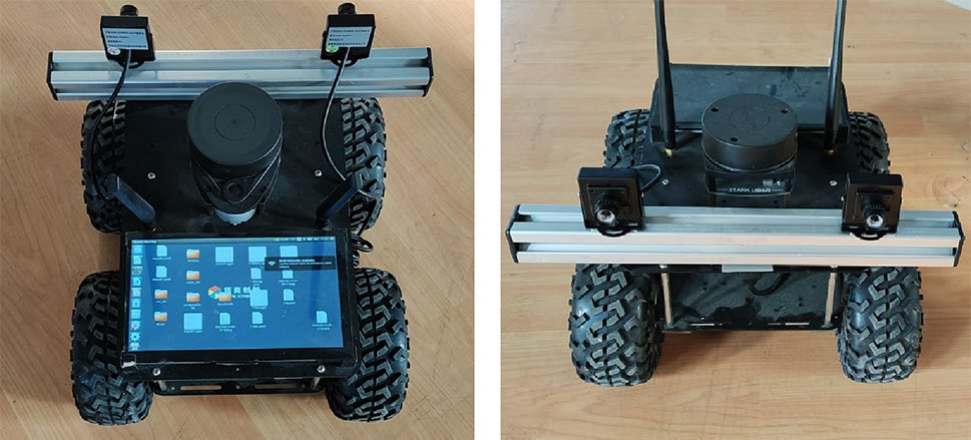
Experimental platform of binocular vision robot.

This section verifies the performance of the algorithm proposed in the article in multiple real-world scenes. The experimental platform used in this article is shown in Fig. 10, consisting of a binocular vision system and a mobile base, with image size collected at 1280$\times$1024 px.

**Figure 11:**
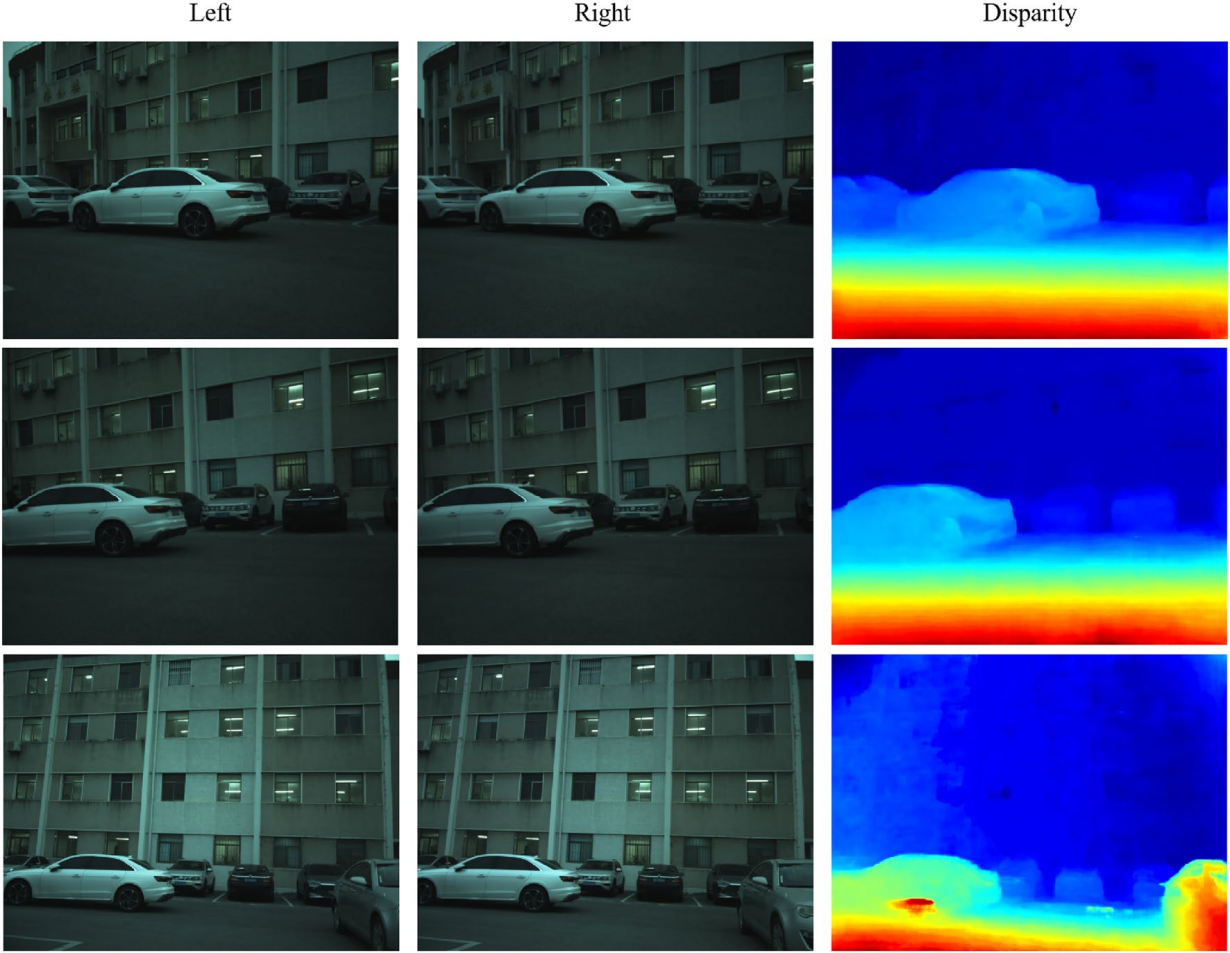
Results in outdoor real-world scenes.

**Figure 12:**
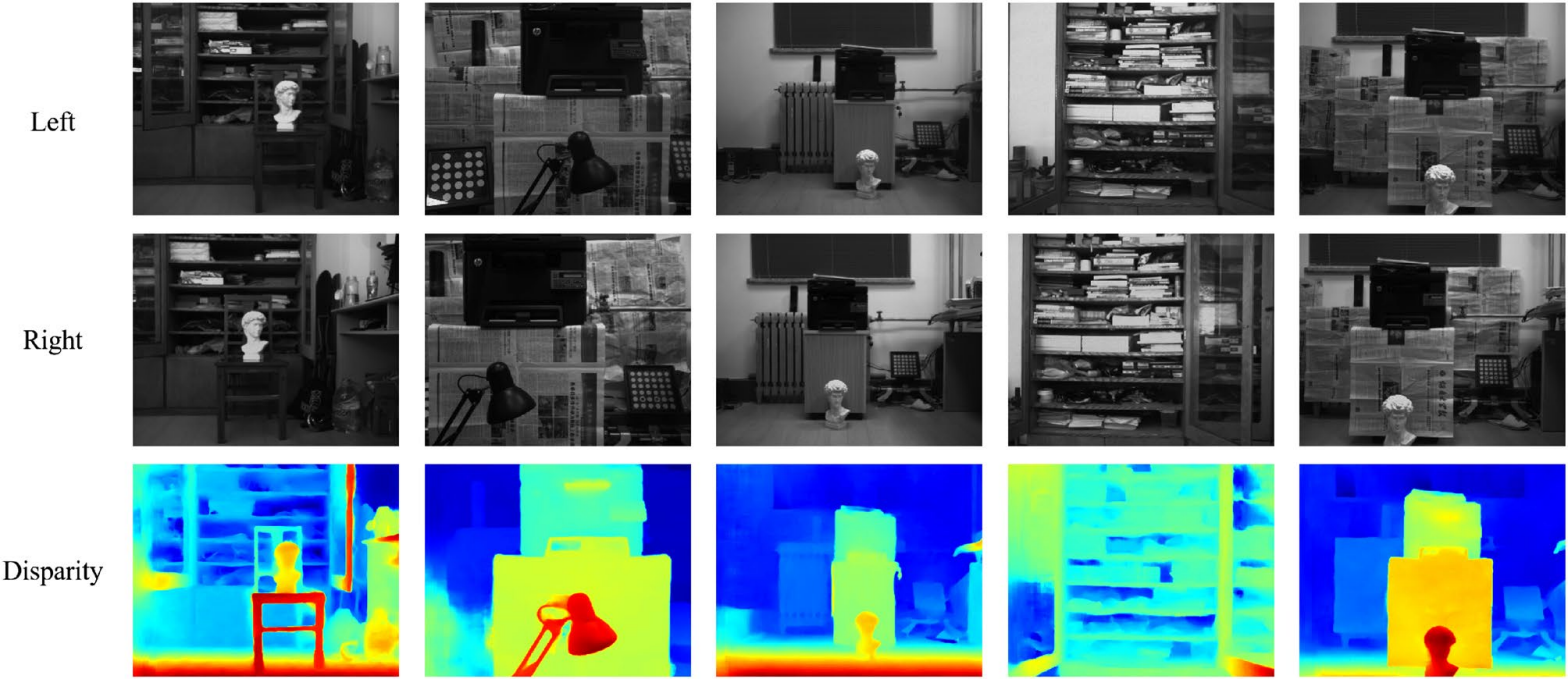
Results in indoor real-world scenes.

The hardware configuration of the car is as follows, it includes a pair of CMOS cameras to form the binocular camera system, which is used to obtain images from the left and right sides, and the camera takes 10 frames per second. In addition, the car uses an embedded processor, the operating system is Ubuntu 18.04, the processor is NVIDIA Jetson Nano. For the runtime environment, the algorithm is invoked by OpenCV for C++ to perform binocular stereo matching. In addition, the car is independently powered by a lithium battery. Among them, the distance measurement algorithm includes stereo matching algorithm, distance measurement and map modeling. Capture images and generate disparity maps in multiple indoor and outdoor scenes using this device. Since indoor disparity is usually higher than outdoor disparity, we take the maximum indoor disparity as 256 and the maximum outdoor disparity as 192. The results of outdoor experiments are presented in Fig. 11, while the results of indoor experiments are shown in Fig. 12.

It is worth noting that our cross-domain stereo matching model predicts disparity directly in the real scene without retraining, so this can test the cross-domain capability of our model. The experimental results of generating disparity maps in various real-world indoor and outdoor scenes demonstrate that the stereo matching algorithm proposed in this article exhibits valuable cross-domain generalization ability, and can satisfy the requirements of completing various tasks in robot vision.

## Conclusion

Computer vision plays a crucial role in enabling robots to acquire depth information of objects and accomplish tasks by simulating the human visual system. This paper proposed a stereo matching network based on transfer learning for domain adaptive stereo matching tasks in robotics. The model is specifically designed to cater to requirements of robots in multiple scenes, and a comprehensive training strategy is formulated to train the network effectively. Furthermore, a general feature extractor is introduced to obtain general feature information, and an adapter is designed to adapt general features to a cost-optimized model of the network. To reduce the domain shift problem, an adaptive disparity optimization module is proposed in this paper to update disparity in stages. Compared with the prototype PSMNet, on KITTI 2015 benchmark, the $3PE-fg$ of Ct-Net in all regions and non-occluded regions decreased by 19.3% and 21.1% respectively, and on the Middlebury dataset, the proposed algorithm improves the sample error rate at least 28.4%, which is the Staircase sample. Experiments on multiple datasets show that the proposed algorithm and training strategy can improve the cross-domain performance of stereo matching.

Our future research will focus on improving the generalization ability of the algorithm and conducting experiments in various domains. Specifically, we plan to integrate the attention mechanism of the Transformer to enhance the matching accuracy and explore the potential of the segmentation task to optimize the matching result of ill-posed regions. Ultimately, we aim to apply the proposed algorithm to an even wider range of real-world scenes.

## Data Availability

Data is contained within the article. The data presented in this study are available in this article.

## References

[1] Suthakorn, J. *et al.* Stereo vision-based object detection and depth estimation from 3d reconstructed scene for an autonomous multi robotic rescue mission (2022).

[2] Li, P., Su, S. & Zhao, H. Rts3d: Real-time stereo 3d detection from 4d feature-consistency embedding space for autonomous driving. *Cornell University–arXiv* (2020).

[3] Zhao H, Wu B (2022). Three-dimensional face modeling technology based on 5G virtual reality binocular stereo vision. Int. J. Commun. Syst..

[4] Du S (2022). A comprehensive survey: Image deraining and stereo-matching task-driven performance analysis. IET Image Process..

[5] Zaidi SSA (2022). A survey of modern deep learning based object detection models. Digital Signal Process..

[6] Li J, Huang X, Tu L (2022). WHU-OHS: A benchmark dataset for large-scale hersepctral image classification. Int. J. Appl. Earth Observ. Geoinform..

[7] He, K., Zhang, X., Ren, S. & Sun, J. Deep residual learning for image recognition. In *2016 IEEE Conference on Computer Vision and Pattern Recognition (CVPR)*, 770–778, 10.1109/CVPR.2016.90 (2016).

[8] Ling Z (2021). Domain-adaptive modules for stereo matching network. Neurocomputing.

[9] Yang, G., Zhao, H., Shi, J., Deng, Z. & Jia, J. SegStereo: Exploiting Semantic Information for Disparity Estimation. *arXiv e-prints* arXiv:1807.11699, 10.48550/arXiv.1807.11699 (2018). 1807.11699.

[10] Xu, G., Cheng, J., Guo, P. & Yang, X. Attention concatenation volume for accurate and efficient stereo matching. In *2022 IEEE/CVF Conference on Computer Vision and Pattern Recognition (CVPR)*, 12971–12980, 10.1109/CVPR52688.2022.01264 (2022).

[11] Rao Z (2020). Nlca-net: A non-local context attention network for stereo matching. APSIPA Trans. Signal Inf. Process..

[12] Wu, Z., Wu, X., Zhang, X., Wang, S. & Ju, L. Semantic stereo matching with pyramid cost volumes. In *2019 IEEE/CVF International Conference on Computer Vision (ICCV)*, 7483–7492, 10.1109/ICCV.2019.00758 (2019).

[13] Chen S, Xiang Z, Qiao C, Chen Y, Bai T (2021). Pgnet: Panoptic parsing guided deep stereo matching. Neurocomputing.

[14] Shen, Z., Dai, Y. & Rao, Z. Cfnet: Cascade and fused cost volume for robust stereo matching. In *2021 IEEE/CVF Conference on Computer Vision and Pattern Recognition (CVPR)*, 13901–13910, 10.1109/CVPR46437.2021.01369 (2021).

[15] Rao Z, Dai Y, Shen Z, He R (2022). Rethinking training strategy in stereo matching. IEEE Trans. Neural Netw. Learn. Syst..

[16] Sang H, Wang Q, Zhao Y (2019). Multi-scale context attention network for stereo matching. IEEE Access.

[17] Shankar, K., Tjersland, M., Ma, J., Stone, K. & Bajracharya, M. A Learned Stereo Depth System for Robotic Manipulation in Homes. *arXiv e-prints* arXiv:2109.11644, 10.48550/arXiv.2109.11644 (2021).

[18] Yang J, Li D, Waslander SL (2021). Probabilistic multi-view fusion of active stereo depth maps for robotic bin-picking. IEEE Robot. Autom. Lett..

[19] Lajoie, P.-Y. & Beltrame, G. *Swarm-slam: Sparse decentralized collaborative simultaneous localization and mapping framework for multi-robot systems* **2301**, 06230 (2023)

[20] Yang X (2022). A novel stereo image self-inpainting network for autonomous robots. Robot. Autonom. Syst..

[21] Shim S, Lee S-W, Cho G-C, Kim J, Kang S-M (2023). Remote robotic system for 3d measurement of concrete damage in tunnel with ground vehicle and manipulator. Comput. Aid. Civ. Infrastruct. Eng..

[22] Obasekore H, Fanni M, Ahmed SM, Parque V, Kang B-Y (2023). Agricultural robot-centered recognition of early-developmental pest stage based on deep learning: A case study on fall armyworm (spodoptera frugiperda). Sensors.

[23] Xiang L (2023). Field-based robotic leaf angle detection and characterization of maize plants using stereo vision and deep convolutional neural networks. J. Field Robot..

[24] Hirschmüller H, Innocent PR, Garibaldi JM (2002). Real-time correlation-based stereo vision with reduced border errors. Int. J. Comput. Vis..

[25] Kolmogorov, V. & Zabih, R. Computing visual correspondence with occlusions using graph cuts. *Proc. Eighth IEEE International Conference on Computer Vision. ICCV 2001* vol. 2, 508–515 (2001).

[26] Sun J, Zheng N-N, Shum H-Y (2003). Stereo matching using belief propagation. IEEE Trans. Pattern Analy. Mach. Intell..

[27] Hirschmuller, H. Accurate and efficient stereo processing by semi-global matching and mutual information. In *2005 IEEE Computer Society Conference on Computer Vision and Pattern Recognition (CVPR’05)*, vol. 2, 807–814, 10.1109/CVPR.2005.56 (2005).

[28] Žbontar, J. & LeCun, Y. Computing the stereo matching cost with a convolutional neural network. In *2015 IEEE Conference on Computer Vision and Pattern Recognition (CVPR)*, 1592–1599, 10.1109/CVPR.2015.7298767 (2015).

[29] Luo, W., Schwing, A. G. & Urtasun, R. Efficient deep learning for stereo matching. In *2016 IEEE Conference on Computer Vision and Pattern Recognition (CVPR)*, 5695–5703, 10.1109/CVPR.2016.614 (2016).

[30] Güney, F. & Geiger, A. Displets: Resolving stereo ambiguities using object knowledge. In *2015 IEEE Conference on Computer Vision and Pattern Recognition (CVPR)*, 4165–4175, 10.1109/CVPR.2015.7299044 (2015).

[31] Kendall, A. *et al.* End-to-end learning of geometry and context for deep stereo regression. In *2017 IEEE International Conference on Computer Vision (ICCV)*, 66–75, 10.1109/ICCV.2017.17 (2017).

[32] Chang, J.-R. & Chen, Y.-S. Pyramid stereo matching network. In *2018 IEEE/CVF Conference on Computer Vision and Pattern Recognition*, 5410–5418, 10.1109/CVPR.2018.00567 (2018).

[33] Guo, X., Yang, K., Yang, W., Wang, X. & Li, H. Group-wise correlation stereo network. In *2019 IEEE/CVF Conference on Computer Vision and Pattern Recognition (CVPR)*, 3268–3277, 10.1109/CVPR.2019.00339 (2019).

[34] Tulyakov, S., Ivanov, A. & Fleuret, F. Practical deep stereo (pds): Toward applications-friendly deep stereo matching. In Neural Information Processing Systems, (2018).

[35] Zhang Y, Li Y, Kong Y, Liu B (2020). Attention aggregation encoder-decoder network framework for stereo matching. IEEE Signal Process. Lett..

[36] Song X, Zhao X, Fang L, Hu H, Yu Y (2019). Edgestereo: An effective multi-task learning network for stereo matching and edge detection. Int. J. Comput. Vis..

[37] Tankovich, V. *et al.* Hitnet: Hierarchical iterative tile refinement network for real-time stereo matching. *CoRR***abs/2007.12140** (2020).

[38] Hu, J., Shen, L. & Sun, G. Squeeze-and-excitation networks. *2018 IEEE/CVF Conference on Computer Vision and Pattern Recognition* 7132–7141 (2017).

[39] Vaswani A (2017). Attention is All You Need. Advances in Neural Information Processing Systems.

[40] Li, J. *et al.* Practical stereo matching via cascaded recurrent network with adaptive correlation. *2022 IEEE/CVF Conference on Computer Vision and Pattern Recognition (CVPR)* 16242–16251 (2022).

[41] Park IK (2018). Deep self-guided cost aggregation for stereo matching. Pattern Recognit. Lett..

[42] Cheng X, Wang P, Yang R (2018). Learning depth with convolutional spatial propagation network. IEEE Trans. Pattern Anal. Mach. Intell..

[43] Cheng, X. *et al.* Hierarchical neural architecture search for deep stereo matching. *ArXiv***abs/2010.13501** (2020).

[44] Zhang F, Vedaldi A, Bischof H, Brox T, Frahm J-M (2020). Domain-Invariant Stereo Matching Networks. Computer Vision-ECCV 2020.

[45] Krizhevsky A, Sutskever I, Hinton GE (2012). Imagenet classification with deep convolutional neural networks. Commun. ACM.

[46] Jeon S, Heo Y (2022). Efficient multi-scale stereo-matching network using adaptive cost volume filtering. Sensors.

[47] Jia, X. *et al.* Multi-scale cost volumes cascade network for stereo matching. In *2021 IEEE International Conference on Robotics and Automation (ICRA)*, 8657–8663, 10.1109/ICRA48506.2021.9560864 (IEEE Press, 2021).

[48] Zhu, Z., Guo, W., Chen, W., Li, Q. & Zhao, Y. Mpanet: Multi-scale pyramid aggregation network for stereo matching. In *2021 IEEE International Conference on Image Processing (ICIP)*, 2773–2777, 10.1109/ICIP42928.2021.9506705 (2021).

[49] Zhong, Y., Dai, Y. & Li, H. Self-Supervised Learning for Stereo Matching with Self-Improving Ability. *arXiv e-prints* arXiv:1709.00930, 10.48550/arXiv.1709.00930 (2017). 1709.00930.

[50] Mayer, N. *et al.* A large dataset to train convolutional networks for disparity, optical flow, and scene flow estimation. In *2016 IEEE Conference on Computer Vision and Pattern Recognition (CVPR)*, 4040–4048, 10.1109/CVPR.2016.438 (2016).

[51] Geiger, A., Lenz, P. & Urtasun, R. Are we ready for autonomous driving? the kitti vision benchmark suite. In *2012 IEEE Conference on Computer Vision and Pattern Recognition*, 3354–3361, 10.1109/CVPR.2012.6248074 (2012).

[52] Scharstein, D. *et al.* High-resolution stereo datasets with subpixel-accurate ground truth. In *German Conference on Pattern Recognition* (2014).

[53] Huang X (2020). The apolloscape open dataset for autonomous driving and its application. IEEE Trans. Pattern Anal. Mach. Intell..

[54] Xu, H. & Zhang, J. Aanet: Adaptive aggregation network for efficient stereo matching. *2020 IEEE/CVF Conference on Computer Vision and Pattern Recognition (CVPR)* 1956–1965 (2020).

[55] Wang H, Fan R, Cai P, Liu M (2021). Pvstereo: Pyramid voting module for end-to-end self-supervised stereo matching. IEEE Robot. Autom. Lett..

[56] Yang, G., Manela, J., Happold, M. & Ramanan, D. Hierarchical deep stereo matching on high-resolution images. In *2019 IEEE/CVF Conference on Computer Vision and Pattern Recognition (CVPR)*, 5510–5519, 10.1109/CVPR.2019.00566 (2019).

[57] Wang, Q., Shi, S., Zheng, S., Zhao, K. & Chu, X. Fadnet: A fast and accurate network for disparity estimation. In *2020 IEEE International Conference on Robotics and Automation (ICRA)*, 101–107, 10.1109/ICRA40945.2020.9197031 (2020).

[58] Liang, Z. *et al.* Learning for disparity estimation through feature constancy. In *2018 IEEE/CVF Conference on Computer Vision and Pattern Recognition*, 2811–2820, 10.1109/CVPR.2018.00297 (2018).

